# Decreased IGF-1 level is associated with restrained amino acid metabolism in NSCLC with diabetes mellitus

**DOI:** 10.3389/fendo.2022.1031798

**Published:** 2022-10-18

**Authors:** Hehe Lv, Fan Zhang, Can Liang, Xuekui Liu, Yamei Ma, Jiayi Li, Yan Ye, Shanwen Si, Yaran Liu, Hao Heng, Houfa Geng

**Affiliations:** ^1^ Department of Endocrinology, Graduate School of Bengbu Medical College, Bengbu, China; ^2^ Department of Endocrinology, Xuzhou Central Hospital, Affiliated Clinical Hospital of Xuzhou Medical University, Xuzhou, Jiangsu, China; ^3^ Department of Medical Examination Center, Xuzhou Central hospital, Xuzhou, China; ^4^ Institute of Medical Artificial Intelligence, Binzhou Medical College, Yantai, China

**Keywords:** diabetes mellitus, NSCLC, amino acid metabolism, untargeted metabolomics, IGF-1

## Abstract

The discovery of a large number of small pulmonary nodules and early diagnosis of lung cancer in the diabetic patients prompt us to re-examine the relationship between diabetes and the occurrence and development of lung cancer. The aim of this study was to explore the underlying metabolites changes in diabetes with NSCLC or benign nodule patients, and further to investigate the association of serum IGF-1 level and differentially expressed metabolites (DEMs). An untargeted metabolomics method was used to detect the changes of metabolism in diabetic patients with NSCLC on the platform of HR-MS. Serum level of IGF-1 was measured by ELISA. The patients were divided to three groups, DM, DLB (nodule), and DLC (cancer). we have identified numerous DEMs, which include amino acid, choline, and fatty acid derivatives. Further analysis of the involved metabolic pathways suggested that linoleate metabolism, tryptophan metabolism, histidine metabolism, putative anti-Inflammatory metabolites formation from EPA, and arachidonic acid metabolism were considered to be the most significant metabolic pathways between groups. Networks analysis suggested that a series of metabolites were associated with serum IGF-1among the three groups, which can be divided into 6 categories. Nine metabolites have been identified as the main DEMs among the DLC, DLB, and DM groups. In conclusion, metabolomics is a powerful and promising tool for the cancer risk evaluation in diabetic patients. Our results suggest that decreased IGF-1 level is associated with restrained amino acid metabolism in NSCLC with diabetes mellitus.

## Introduction

The IDF Diabetes Atlas estimates that the global prevalence of diabetes mellitus (DM) is over 10% in 2021 (1), while the prevalence of diabetes in China adults has already exceed the world average level and reached 11.2% using the WHO criteria in 2018 (2). Based on the largest population, the number of diabetes patients will be over 156 million nowadays. As is well known, diabetes could result in the increased risk of morbidity and total mortality due to its complications and comorbidities, which include cardiovascular disease, stroke, and cancer, etc. A series of studies have suggested the association between diabetes mellitus and various kinds of cancer. Even the results were not always consistent, there is emerging evidence showed that DM is accompanied with increased cancer-specific mortality (3).

According to the Global Cancer Statistics 2020 (4), lung cancer still remained the leading cause of cancer death and the incidence ranks the second with estimated 2.2 million new cases (11.4%), of which the majority is Non-Small Cell Lung Cancer (NSCLC). A large sample retrospective analysis in Japan showed that the cancer risk with diabetes was both increased in males and females significantly, OR= 1.44 (1.28-1.62) and 1.39 (1.19-1.62), respectively (5). Among which the incidence rate of lung cancer in diabetes patients increased by 53% in males and 61% in females, after correcting the confounding factors, such as age, obesity, smoking and other risk factors. A meta-analysis showed that there is no positive association between diabetes and lung cancer (RR = 0.99, 0.88-1.11), but after correcting smoking status, diabetes could increase the risk of lung cancer by 11% (RR = 1.11, 1.02-1.20) (6). Diabetes is also an independent risk factor in NSCLC patients, of which the risk of all-cause death is 69% higher than that of non-diabetes patients (HR=1.69, 1.25-2.30). Furthermore, perioperative blood glucose control significantly improves the overall survival rate of lung cancer (HR = 0.621, 0.42-0.92) (7).

However, the underlying mechanism has been constantly explored. Many factors have been reported to be involved in the development and progression of cancer in DM, including hyperinsulinemia (8), elevated free insulin-like growth factor-1 (IGF-1) level (9), impaired IGF binding protein (IGFBP)/IGF/IGF receptor (IGFR) axis, and inflammatory cytokines (10). Yu (11) has reported that elevated serum levels of IGF-1 and decreased IGFBP-3 levels were positively associated with the risk of lung cancer. IGF-1 is one kind of cell proliferation regulator with 40% homology with insulin precursor, including IGF-1 and IGF-2, which usually exists in the form of dimer or trimer formed by binding with IGFBP and plays its role by binding with IGFR. IGF-1R is one essential member of the tyrosine kinase growth factor receptor super-family. The combination of IGF-1 and IGF-1R can activate the PI3K and MAPK signaling pathways, thereby promoting the proliferation, metastasis and anti-apoptosis of tumor cells (12). Insulin can increase the level of serum free IGF-1 through the inhibition of IGFBP-1 production (13). Most patients with diabetes have hyperinsulinemia, IGFBP is suppressed to a certain extent, and the level of free IGF-1 is relatively high. Researches (12) have shown that IGF-1 level is increased in many site-specific cancers and could activate the signal pathway to promote the development of cancer, including lung, colorectal, breast, and prostate. IGF-1 and fibroblast growth factor -1 (FGF-1) can synergistically promote the expansion of lung cancer stem cells (LCSCs) and significantly down regulate the apoptotic signals through the activation of MAPK signaling pathway (14). However, Qian (15) has reported positive associations of IGF-1 with breast, prostate, colorectum, melanoma, kidney, thyroid, and inverse associations with lung cancer in ever-smokers. As the previous research results are inconsistent, further research is needed to explore the true relationship.

With the continuous prevalence of COVID-19, chest CT has become an ordinarily screening program for diabetic patients, followed by the discovery of a large number of small pulmonary nodules and early diagnosis of lung cancer in the clinical practice. Is this just an accidental event, or an inevitable result of diabetes? This problem forced us to re-examine the relationship between diabetes and the occurrence and development of lung cancer. Metabolomics, as a semi-quantitative analysis method of metabolites, can achieve the purpose of quantitative measurement of most endogenous metabolites in a single blood sample (16). Metabolomics has been used to investigate the mechanistic understanding of diabetes and related disease for the last decade (17). In recent years, metabolomics has been widely used in the exploration of the pathogenesis of diabetes, the evaluation of complications and treatment, especially in the synergistic mechanism of traditional Chinese medicine (18). Based on the objectivity, accuracy, and comprehensiveness of metabolomics testing for metabolic evaluation, we can conveniently carry out the correlation between serum biomarkers and clinical results under the condition of diabetes.

In this Study, we conducted the serum IGF-1 analysis and the untargeted metabolomic strategy in the population of diabetes with lung cancer and nodules. This study aimed to highlight the mechanistic insights involved in the association between diabetes and lung cancer.

## Materials and methods

### Study subjects

**Figure 1:**
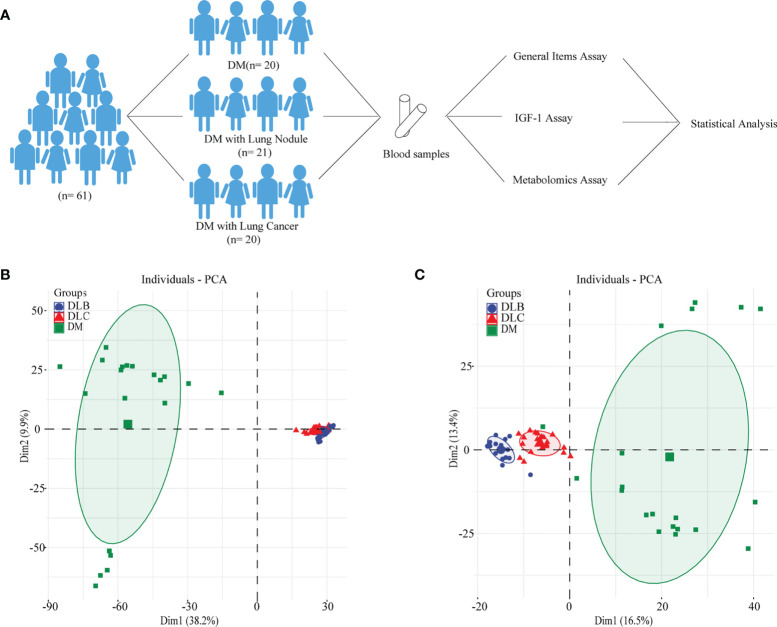
Study Design **(A)**, metabolimics analysis of the whole population using PCA-positive mode **(B)**, metabolimics analysis of the whole population using PCA-negative mode **(C)**.

All the participants enrolled in this study were from the department of Endocrinology and Thoracic Surgery in Xuzhou Central Hospital since April 2019 to July 2021, who were informed of the relevant benefits and risks in writing and voluntarily signed the informed consent. Study population include three groups (**Figure 1A**): 20 type 2 diabetic patients without lung nodule or cancer (DM, n = 20), diabetic patients with lung benign nodule (DLB, n = 21), and diabetic patients with lung cancer (DLC, n = 20).

The main inclusion criteria: Type 2 Diabetes Mellitus, according to the 1999 WHO diagnostic criteria; 40 ≤ age ≤ 80 years old; 18 ≤ Body mass index (BMI) ≤ 30 kg/m^2; Diabetic patients with no abnormal pulmonary CT were taken as the DM group; Diabetic patients with small lung nodules but no malignant signs, were enrolled in the DLB group; For those with malignant signs, such like irregular edge, burr sign, halo sign, calcification, ground glass density, and infiltrative growth, who eventually undertaken surgical resection and confirmed as NSCLC by pathology, were taken as DLC group. Exclusion criteria: Patients with a past history of cancer, pneumonia and any other site’s acute inflammation, hypertension, acute heart infarction, severe hepatic lesion, renal dysfunction, severe anemia, and other serious situations.

### Anthropometric measurements and laboratory methods

Anthropometric measurements were obtained from a clinical questionnaire, including age, gender, age, smoking status, and other basal information. Weight and height were measured in kg and cm, respectively. BMI was calculated as weight (kg)/height^2 (m^2). Blood pressure measurement was taken by a physician using a mercury sphygmomanometer, which was after 30 minutes rest at least.

Blood samples were collected after fasting for at least 10 hours and used for the detection of hepatic function, renal function, lipid profile, fasting blood glucose (FBG), and 2 hours postprandial blood glucose (2hPG). All of the measurements were taken at the clinical laboratory of Xuzhou Central Hospital. Serum samples for the metabolomics analysis were frozen at -80°C. An ELISA KIT (Beyotime, Nanjing, China) was used to assay the serum levels of IGF-1. The detection process refers to the instructions of ELISA kit. The standard sample shall be diluted. After adding the sample and enzyme, incubate for 10 minutes at 37°C. Finally, the OD value will be obtained at 450nm by using the microplate reader.

### High performance liquid chromatography-mass spectrometry (HPLC-MS) detection

Serum samples were pretreated before detection by HPLC-MS. In this process, 200ul serum was taken, 1200 μ L of precooled methanol and acetonitrile were used for the sample protein precipitation. After 10 minutes standing, the mixture was centrifuged at 12000g for 10 minutes. The supernatant was volatilized and dried by rotary evaporation to obtain dry powder. Finally, acetonitrile (50μ L) was used to dissolve the dry powder.

LC separation was performed on the Waters E2695 Ultra HPLC (Waters, USA) platform using the Fortis type C18 column (2.1×100 mm, 1.7 μm) at a column temperature of 40°C. Water/acetic acid (99.9/0.1, v/v) is used as solvent A and methanol is used as solvent B in the mobile phase. During the injection, take 5 ul sample volume. Gradient is 0 min, 95% (A); 1 min, 75% (A); 3 min, 55% (A); 6 min, 5% (A); 12 min, 5% (A). The post-treatment time is 2 min with a flow rate of 0.3 ml/min. Positive and negative modes for performing MS analysis in full scan mode on the Thermo HFX mass spectrometer (Thermo, CA, USA). The solution is injected into an ESI source and then at a rate of 0.3 ml/min, capillary of 4000 V, dry gas of 12 L/min, and dry gas temperature of 350°C. Optimization of the MS conditions was repeated for maximum detection sensitivity. Prior to sample analysis, in order to guarantee system stability, quality control (QC) samples were tested 6 times and mixed with all serum samples.

### Data statistics

The deconvolution of LC-MS spectrum was analyzed by Thermo Data Analysis software, including baseline correction, noise process, and peak alignment. Retention time (m/z) formed matrix analysis was performed by compound detection. R4.1.3 software was preformed to analyze the metabolites data from LC-MS. The principal component analysis (PCA) was used to show the difference among three groups (19), and then orthogonal PLS-DA (OPLS-DA) and t-test were performed to explore the differential expressed metabolites (DEMs) between two groups. The R packages of “ropls” were used to divide the samples into two groups. Heat map and volcano plot were used to show the DEMs between two groups. The Metaboanalyst web was used to find the DEMs related pathway (20). DEMs among the three groups were further analyzed by One-way ANOVA.

## Results

### General characteristics of the study population

The basic clinical characteristics of the study subjects were presented in **Table 1**. This study had 38 males and 23 females. Smokers made up 40.98% (25/61) of the study participants. There was no statistical difference among the three groups in the general items, such as sex, age, smoking status, BMI, HbA1c, lipid profile, etc. Serum IGF-1 level in the group of DM was significantly higher than that of DLB and DLC.

**Table 1:** General characteristics of the study population.

Characteristics (Means ± SD)	Diabetes Mellitus (n=20)	Diabetes with Lung Nodule (n=21)	Diabetes with Lung Cancer (n=20)	P Value
Sex (Male)	13	14	11	0.710
Age(years)	63.25±12.02	61.50±11.83	68.57±6.73	0.092
Course of DM (years)	5.25±3.28	4.44±2.09	6.32±2.01	0.116
Smoking (n)	7	9	9	0.540
BMI (kg/m^2)	24.77±5.21	24.82±3.53	24.83±5.45	0.991
FPG (mmol/L)	9.87±6.14	8.96±3.03	10.51±8.17	0.718
2hPG(mmol/L)	15.67±4.32	16.31±6.17	15.89±3.88	0.914
HbA1c (%)	9.23±4.35	8.25±2.76	10.05±3.55	0.284
TC (mmol/L)	4.33±0.84	4.23±0.95	4.25±1.20	0.946
TG (mmol/L)	1.54±0.73	1.23±0.69	1.17±0.84	0.257
ALT(U/L)	49.77±12.38	50.12±19.23	48.96±21.33	0.978
AST(U/L)	77.15±24.51	81.06±33.11	79.59±29.47	0.912
eGFR (ml/min)	81.34±16.99	79.87±21.42	82.05±32.61	0.959
IGF-1 (ng/ml)	181.25±24.88	160.40±17.33	158.68±19.47	<0.001

BMI, body mass index; FPG, fasting plasma glucose; 2h PG, 2 hours plasma glucose; HbA1c, glycosylated hemoglobin; TC, total cholesterol; TG, triglyceride; ALT, alanine aminotransferase; AST, glutamic oxalacetic transaminase; eGFR, estimated glomerular filtration rate; IGF-1, Insulin-like growth factor 1.

### Untargeted Metabolomics Analysis of the participants

To ensure the stability of our HPLC-MS system, we both conducted the positive ion mode and the negative ion mode. The QC samples results suggested to be clustered with a small relative standard deviation (RSD) of different peaks, which showed the stability of our HPLC-MS system. The raw MS data in the Thermo Data Analysis software was used to establish data matrix and then analyzed by OPLS-DA and PCA. As shown in the **Figures 1B, C**, PCA score plot suggested that serum samples of the DLC could be clearly discriminated from DLB and DM group.

**Figure 2:**
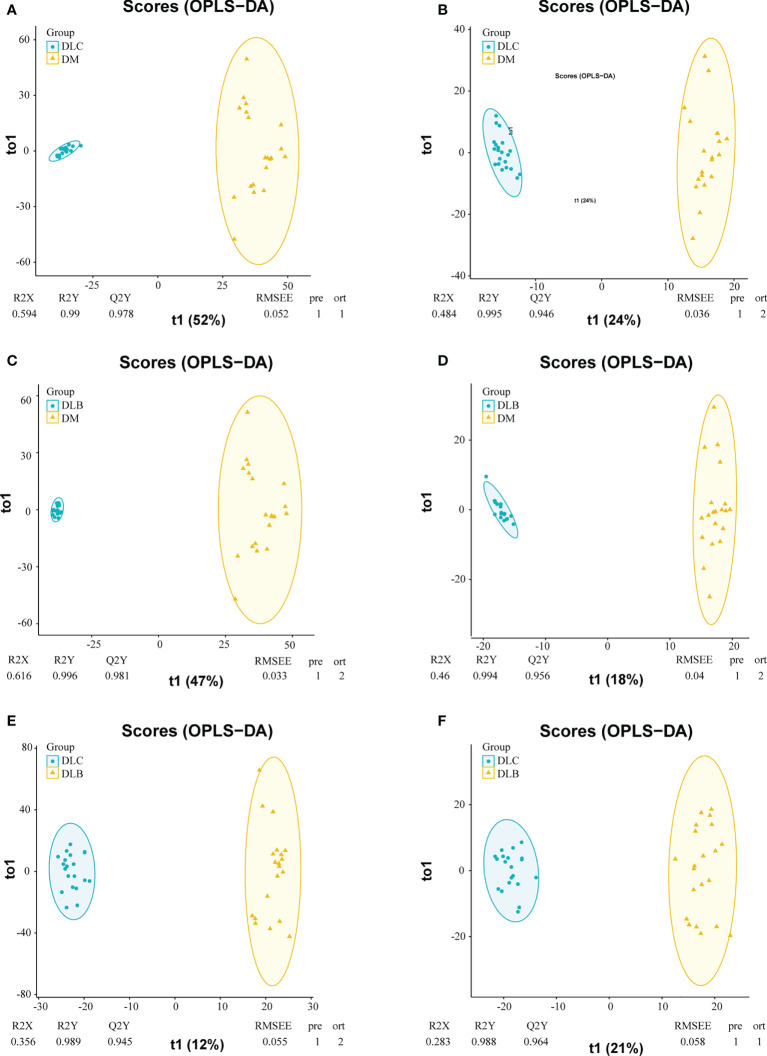
OPLS-DA plots of the metabolities detected in serums, DLC vs DM in positive mode **(A)**, DLC vs DM in negative mode **(B)**; DLB in positive mode **(C)** DLB vs DM in negative mode **(D)** DLC vs DLB in positive mode **(E)**, DLC vs DLB in negative mode **(F)**.

OPLS-DA was further conducted to investigate the DEMs between each two groups. Serum samples were clearly distinguished from groups either in the positive mode or in the negative mode. The Q2Y and R2Y values between the diabetes group and diabetes with lung cancer group were 0.978 and 0.990, 0.946 and 0.995, respectively (**Figures 2A, B**). The Q2Y and R2Y values between the diabetes group and diabetes with lung nodule group were 0.981 and 0.996, 0.956 and 0.994, respectively (**Figures 2C, D**). And the Q2Y and R2Y values between the nodule group and cancer group were 0.945 and 0.989, 0.9645 and 0.988, respectively (**Figures 2E, F**).

**Figure 3:**
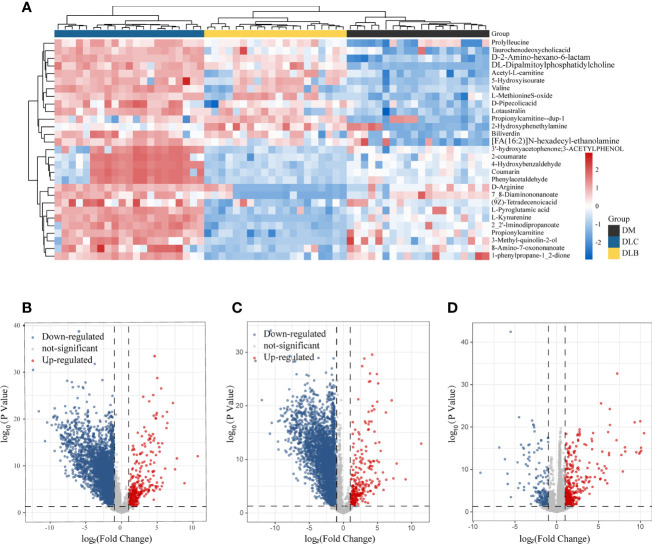
Differential metabolities, The heatmap illustrating the levels of DEMs among the three groups **(A)**, Volcano plot showing the DEMSs between DLC and DM **(B)**, the DEMs between DLB and DM **(C)**, THE demS between DLC and DLB **(D)**.

Based on the database KEGG and inhouse reference metabolites, Thermo Data Analysis software was used to annotate the peaks of the LC-MS chromatograms. To identify the DEMs among the three groups, further analysis of those metabolites with fold change >2 or <0.5 was performed. There were 3605 down-regulated and 328 up-regulated metabolites between diabetes and cancer groups (**Figures 3A, B**), 3871 down-regulated and 285 up-regulated metabolites between diabetes and nodule groups (**Figures 3A, C**), and 205 down-regulated and 426 up-regulated metabolites between nodule and cancer groups (**Figures 3A, D**). In short, we have identified numerous DEMs, which include amino acid, choline, and fatty acid derivatives, such as prolyl leucine, taurochenodeoxycholicacid, D-2-Amino-hexano-6-lactam, DL-Dipalmitoylphosphatidylcholine, Acetyl-L-carnitine, 5-Hydroxyisourate, valine, and biliverdin, etc.

**Figure 4:**
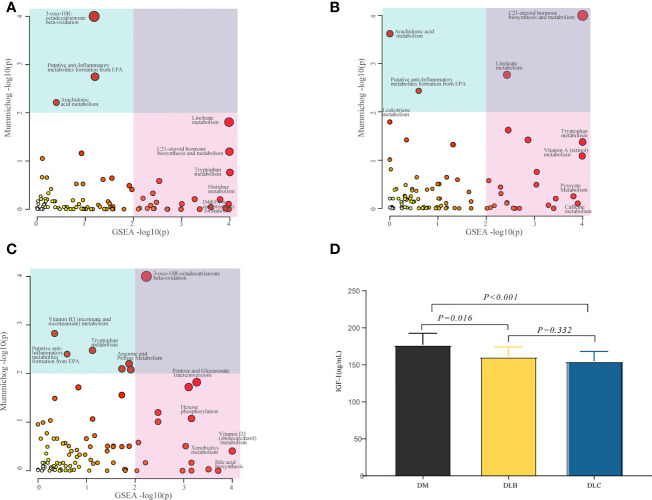
Differential metabolic pathway, The bubble plot showing the enriched pathways of DEMs between DLC and DM **(A)**, the enriched pathways of the DEMs between DLB and DM **(B)**, the enriched pathways of the DEMs between DLC and DLB **(C)**; Serum IGF-1 level among the three groups **(D)**.

MetaboAnalyst web tool was used for the metabolic pathway enrichment analysis between two groups, DLC vs DM, DLB vs DM, and DLC vs DLB, separately. Metabolic pathways involved in linoleate metabolism, C21-steroid hormone biosynthesis and metabolism, histidine metabolism, 3-oxo-10R-octadecatrienoate beta-oxidation, tryptophan metabolism, D4&E4-neuroprostanes formation, putative anti-Inflammatory metabolites formation from EPA, and arachidonic acid metabolism were considered to be the most significant metabolic pathways between DLC and DM (**Figure 4A**). Metabolic pathways involved in linoleate metabolism, tryptophan metabolism, arachidonic acid metabolism, putative anti-Inflammatory metabolites formation from EPA, pyruvate metabolism, vitamin A (retinol) metabolism, and caffeine metabolism were considered to be the most significant metabolic pathways between DLB and DM (**Figure 4B**). Metabolic pathways involved in tryptophan metabolism, pentose and glucuronate interconversions, arginine and proline metabolism, hexose phosphorylation, vitamin B3 (nicotinate and nicotinamide) metabolism, bile acid biosynthesis, Vitamin D3 (cholecalciferol) metabolism, and xenobiotics metabolism were considered to be the most significant metabolic pathways between DLC and DLB (**Figure 4C**).

### Changes of IGF-1 level in the participants

As shown in **Figure 4D**, both of the IGF-1 levels in DLC and DLB were decreased compared with DM group, P<0.01 and P< 0.05, respectively. However, there was no statistically significance between DLC and DLB.

### Relationship between serum level of IGF-1 and DEMs

**Figure 5:**
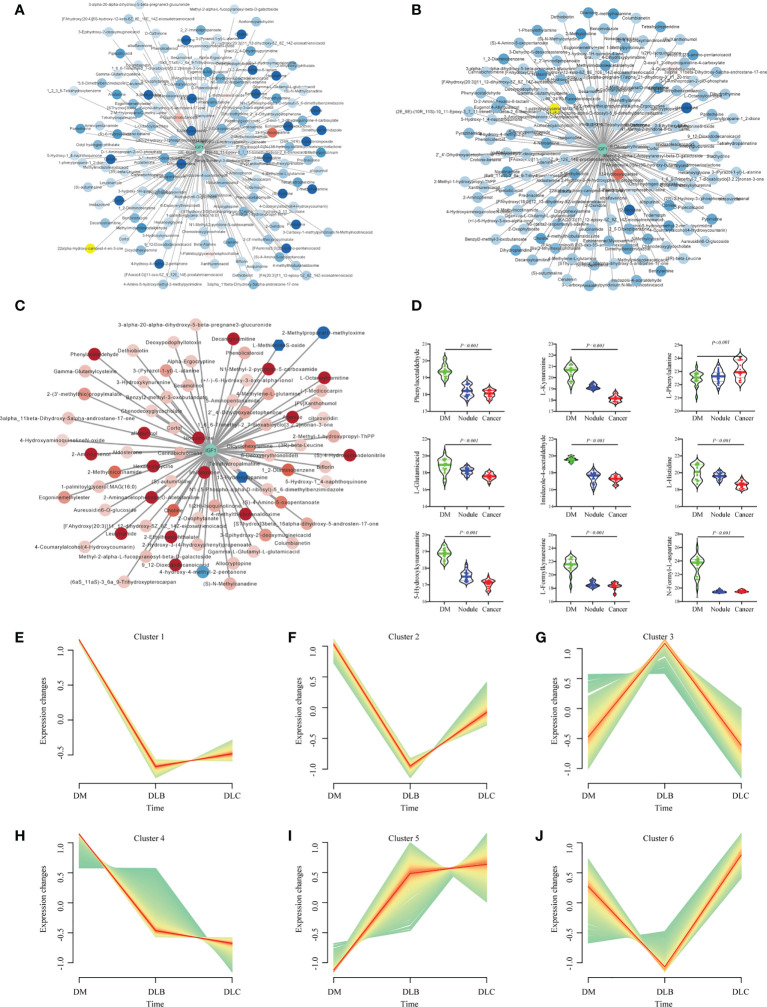
Networks of the IGF-1 and DEMs between DLC and DM **(A)**, Networks of the IGF-1 and DEMs between DLB **(B)**, Networks of the IGF-1 and DEMs between DLC and DLB **(C)**; Representatives of the three groups **(D)**; Different trend cluster of the DEMs among the three groups **(E–J)**.

Networks analysis suggested that a series of metabolites were associated with serum IGF-1 between DLC and DM group, of which the correlation coefficient is > 0.5 or < - 0.5 were shown in **Figure 5A**. Similar results were found during the comparison of DLB vs DM, and DLC vs DLB group (**Figures 5B, C**). According to the expression of metabolites, they can be divided into 6 categories (**Figures 5E–J**). The majority of the related metabolites were amino acids and its derivatives. Combined with the previous analysis, nine metabolites have been identified as the main DEMs among the DLC, DLB, and DM groups, including eight down-regulated metabolites, L-kynurenine, L-glutamic acid, L-histidine, N-Formyl-L-aspartate, phenylacetaldehyde, imidazole-4-acetaldehyde, 5-hydroxykynurenamine, L-formylkynurenine, and one up-regulated metabolite, L-phenylalanine (**Figure 5D**).

## Discussion

According to the general data of the study population, there was no significant difference in the age, sex, smoking status, BMI, course of diabetes, glycated hemoglobin among the group of DM, DLB and DLC. The consistency of baseline ensures the reliability of subsequent analysis.

Since the relationship between serum IGF-1 and cancer was not always in consistency, we performed the detection of IGF-1 concentration in the serum of DM, DLB and DLC. The result comes to be decreased IGF-1 levels in DLC and DLB group compared with DM. Kentaro once reported that serum IGF-1 levels were significantly decreased in Japanese patients with uncontrolled T2DM (21). The participants in this study also have a poorly controlled HbA1c, which partly explains the decreased level of IGF-1. Recently, data from the UK Biobanks suggested that circulating IGF-I concentrations were positively associated with colorectal cancer, breast cancer and prostate cancer, but not associated with NSCLC risk (22). Our result showed an inverse association between DLC and DM, which was inconsistent with UK Biobank outcome, may cause by the diabetic background of the participants in a certain extent.

To further explore the effect of diabetes on the development and progression of NSCLC, untargeted metabolomics analysis was used to examine the metabolite changes in serum of NSCLC and lung benign nodule in diabetes mellitus on the platform of HPLC-MS. In order to get a better distinction, we subjected the mass spectrometry data to OPLS-DA and PCA analyses. Numerous DEMs have been identified in the present study, especially those involved in amino acid metabolism, which is essential for lung cancer cell proliferation and regulation of immune cell function (23, 24). Our results suggested that the metabolomics of DLC was significantly different from DLB and DM.

According to the DEMs, we analyzed the involved metabolic pathways. The linoleate metabolism, tryptophan metabolism, histidine metabolism, putative anti-Inflammatory metabolites formation from EPA, and arachidonic acid metabolism were considered to be the most significant metabolic pathways between group DLC and group DM. Interestingly, we also found the alteration of tryptophan metabolism pathway between DLC and DLB, so does the comparison of DLB and DM, while the tryptophan metabolism pathway was considered as the potential pathway in the pathogenesis of several lines of cancer. Tryptophan can be enzymatically hydrolyzed into kynurenine *in vivo*, through which to participate in cancer immune regulation (25). Our result showed that kynurenine level was downregulated in the DLC group than those of DLB and DM, while Yuzo Suzuki (26) has reported that kynurenine concentrations were significantly higher in lung cancer patients compared with the controls. Recently, Ran Li concluded that no causal relationship was found between kynurenine and lung cancers (27). The downregulation of kynurenine in the DLC group might be associated with decreased IGF-1 level under the condition of diabetes mellitus.

In conclusion, metabolomics is a powerful and promising tool for the cancer risk evaluation in diabetic patient. Our results suggest that decreased IGF-1 level is associated with restrained amino acid metabolism in NSCLC with diabetes mellitus.

## Data availability statement

The original contributions presented in the study are included in the article/Supplementary Materials. Further inquiries can be directed to the corresponding authors.

## Ethics statement

The studies involving human participants were reviewed and approved by The Ethics Committee of Xuzhou Central Hospital. The patients/participants provided their written informed consent to participate in this study.

## Author contributions

HG, HH, and CL designed the experiments and analyzed the data. HG, HL, YL, CL, FZ, YM, JL, SS, YY and XL performed the experiments. HG and YL supervised the study. HG, HL, and FZ wrote the manuscript. All authors contributed to the article and approved the submitted version.

## Funding

This work was supported by Science and Education Project for Young medical talents, Jiangsu Province, China, No. QNRC2016388; The social development project of the Xuzhou Municipal Science and Technology Bureau, No. KC21231.

## Conflict of interest

The authors declare that the research was conducted in the absence of any commercial or financial relationships that could be construed as a potential conflict of interest.

## Publisher’s note

All claims expressed in this article are solely those of the authors and do not necessarily represent those of their affiliated organizations, or those of the publisher, the editors and the reviewers. Any product that may be evaluated in this article, or claim that may be made by its manufacturer, is not guaranteed or endorsed by the publisher.
